# Long-term administration of tacrolimus and everolimus prevents high cholesterol-high fructose-induced steatosis in C57BL/6J mice by inhibiting de-novo lipogenesis

**DOI:** 10.18632/oncotarget.15194

**Published:** 2017-02-08

**Authors:** Sharma Love, Malik A. Mudasir, Subhash C. Bhardwaj, Gurdarshan Singh, Sheikh A. Tasduq

**Affiliations:** ^1^ PK-PD and Toxicology Division, CSIR-Indian Institute of Integrative Medicine, Jammu Tawi, Jammu and Kashmir, India; ^2^ Academy of Scientific and Innovative Research (AcSIR), Chennai, India; ^3^ Department of Pathology, Government Medical College, Jammu, Jammu and Kashmir, India

**Keywords:** NAFLD, tacrolimus, everolimus, mTOR, lipogenesis

## Abstract

**Aim:**

To investigate the effects of tacrolimus (TC) and everolimus (EV) on non-alcoholic steatohepatitis (NASH) induced by high fat, high cholesterol and fructose (fast food) diet in C57BL/6J mice.

**Materials and Methods:**

C57BL/6J mice were divided into four groups (n=8). 1) Standard Chow (SC); 2) Fast food (FF) diet; 3) FF + Tacrolimus (TC, 1mg/kg) and; 4) FF + Everolimus (EV, 1mg/kg) and treated for 16 weeks. Serum and tissue samples were analyzed for evidence of inflammation, fibrosis, lipogenesis, and apoptosis.

**Results:**

TC and EV treatments significantly reduced the hepatic lipid accumulation, improved liver-body weight ratio, blood biochemistry, and insulin resistance in mice fed with FF diet. However, inflammation, enlarged portal tracts, and fibrosis were pronounced in EV treated group. The lipogenic parameters, Peroxisome proliferator-activated receptor gamma (PPAR-γ), Sterol regulatory element-binding protein 1(SREBP-1), mammalian target of rapamycin (m-TOR), Stearoyl-CoA desaturase-1 (SCD-1) and fatty acid translocase (CD36) were significantly down-regulated in livers of TC and EV treated groups as compared to FF group. TC improved Bcl2/Bax ratio, decreased apoptosis, CYP2E1 protein expression and liver fibrosis levels, however, EV offered no such protection. Further, in an *In-vitro* model of lipotoxicity using the mouse hepatocyte (AML-12) cell line, treatment with TC and EV significantly reduced lipid accumulation and lipogenic and apoptotic markers induced with palmitic acid.

**Conclusion:**

In FF diet induced model of NASH, both TC and EV inhibited hepatic lipid accumulation and improved metabolic parameters such as insulin resistance and dyslipidemia. However, mice administered with EV exhibited inflammatory and fibrotic responses despite reduced hepatic steatosis.

## INTRODUCTION

**Figure 1:**
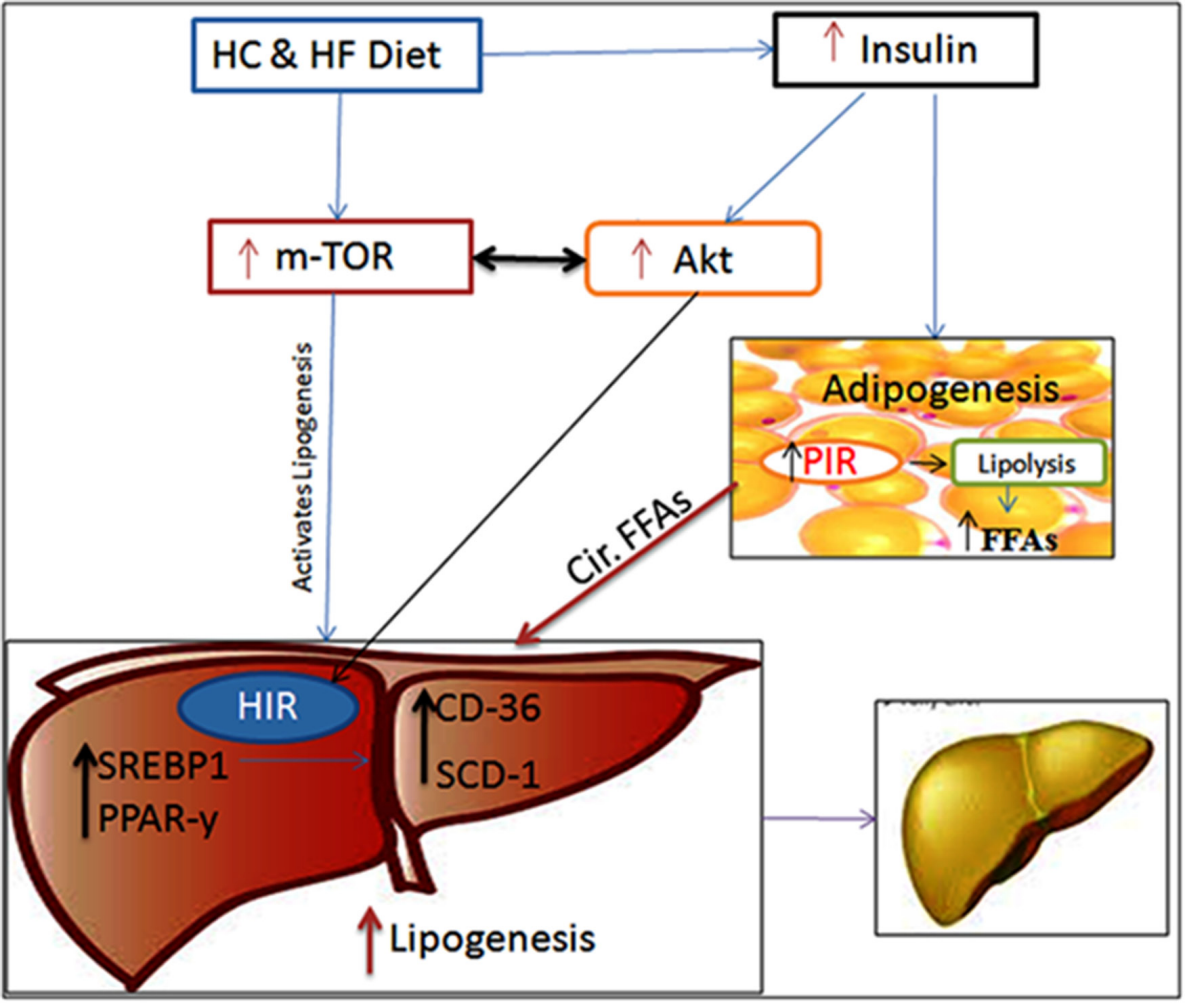
Schematic diagram representing the roles of diet, m-TOR-Akt-insulin signaling in developing NAFLD Diet rich in carbohydrates and fat activates m-TOR signaling, and stimulate insulin release peripherally. m-TOR activation directly up-regulates hepatic lipogenic players SREBP1 and PPAR-γ, thus enhancing the hepatic lipogenesis, via up-regulation of SCD-1 and CD-36. Dysregulation in m-TOR-Akt-insulin signaling induces HIR and PIR. PIR leads to enhanced lipolysis which in turn increase the circulatory load of FFAs towards hepatic circulation, which acts as adjuvant to increased hepatic lipogenesis. All these bio-chemical and molecular events increase the lipid load in hepatocytes leading to NAFLD. Abbreviations: FFA-Free fatty acids, HC & HF – High carbohydrate and high fat, HIR – Hepatic insulin resistance. PIR-Peripheral insulin resistance.

Metabolic derangements such as obesity, type 2 diabetes mellitus (T2DM), dyslipidemia, and related co-morbidities, collectively referred to as metabolic syndrome (MS), create significant socio-economic health burden and is shaping into a global epidemic threat. A major consequent of MS is Non-alcoholic Fatty Liver Disease (NAFLD), whose prevalence has been increasing over the last decade [1]. NAFLD usually manifests clinically, with a range of symptoms from steatosis to non-alcoholic Steatohepatitis (NASH), the latter reflecting inflammation and fibrosis of chronic liver injury [2]. The prevalence of NAFLD in the west is 20-30% and that of NASH is 2-3% [3]. NAFLD pathogenesis is associated with perturbances in several cellular pathways viz phosphoinositide 3-kinase-phosphatase and tensin homolog (PI3K-PTEN), hedgehogg signaling, and mammalian target of rapamycin (m-TOR). Among these, the m-TOR signaling pathway is critically being implicated in senescence, metabolic disorders, aging, cell proliferation and cellular growth [4, 5]. Although it is recognized as key regulator of lipid homeostasis, its role in the pathogenesis of NAFLD is quite controversial. Mammalian target of rapamycin complex 1 (mTORC1), a key component of m-TOR pathway is reported to be involved in the activation of many lipogenic transcriptional factors and proteins, like sterol regulatory element-binding protein 1 (SREBP1), an activator of stearoyl-CoA desaturase-1 (SCD-1) (Figure 1) [6].

Various *In-vivo* models of NAFLD have been developed over the years. These include genetically knockout, surgical and diet-induced models [7]. However, high fat diet and high fructose-fed models have been closely linked with pathogenesis of clinical NAFLD [8]. High fat diet/Western diet fed mice developed insulin resistance (IR), obesity, steatosis, and up-regulation of lipogenic molecular players like PPARs, SREBP1 [9, 10], while treatment with m-TOR inhibitor, rapamycin prevented high fat diet induced obesity [11]. Chronic m-TOR inhibition with rapamycin has resulted in insulin resistance and glucose intolerance, which can also be linked with disruption of mTORC-2 complex [12, 13, 14].

As seen with rapamycin, the effects of calcineurin inhibitor tacrolimus on hepatic steatosis is also obscure. Post liver transplant, clinically it has been confirmed that tacrolimus has a positive association with lipid accumulation in grafted liver, which may be attributed to development of post–transplant diabetes mellitus (PTDM), impaired lipid metabolism and glucose intolerance [15, 16, 17]. However, the mechanisms underlying this phenomenon remain un-explored. According to a report, treatment with tacrolimus had aggravating effects on liver fibrosis in rats [18], whereas others have shown a protective effects of tacrolimus in fibrosis or steatosis. Tacrolimus prevented pulmonary fibrosis, and experimentally induced liver fibrosis [19, 20]. Tacrolimus has been reported to ameliorate ischemia and reperfusion associated liver injury in a mouse model of steatotic necrosis [21]. Over time, steatosis, may progresses to steatohepatitis, of which fibrosis is a clinical outcome and despite therapeutic advances, fibrosis due to NAFLD/NASH induced liver injury remain progressive, while therapies hitting the specific etiologies of NAFLD/NASH, are not available and its prognosis is usually poor, resulting in End-Stage Liver Disease (ESLD) [22]. In such circumstances liver transplantation is the only life saving but costly and uncomfortable option [23]. Despite the demographic importance of NAFLD/NASH, the standard set of therapy and US-FDA approved treatment regimens are lacking and pharmacological interventions have been disappointing [22].

Keeping in view, the controversial background of everolimus and tacrolimus in the prevention of NAFLD, the present study investigated the role of these two drugs i.e. tacrolimus and everolimus in a mouse model of NASH (C57BL/6J mice fed with High Cholesterol/High Fat Diet + High Fructose Corn Syrup {FF, diet}). Both the drugs were administered chronically, over a period of 120 days. To further, complement our *In-vivo* data and explore the in-depth mechanisms involved, immortalized hepatocytes, AML-12, were employed.

## RESULTS

### Chronic administration of tacrolimus and everolimus prevented weight gain, improved altered blood biochemistry in C57BL/6J mice fed on FF

**Figure 2:**
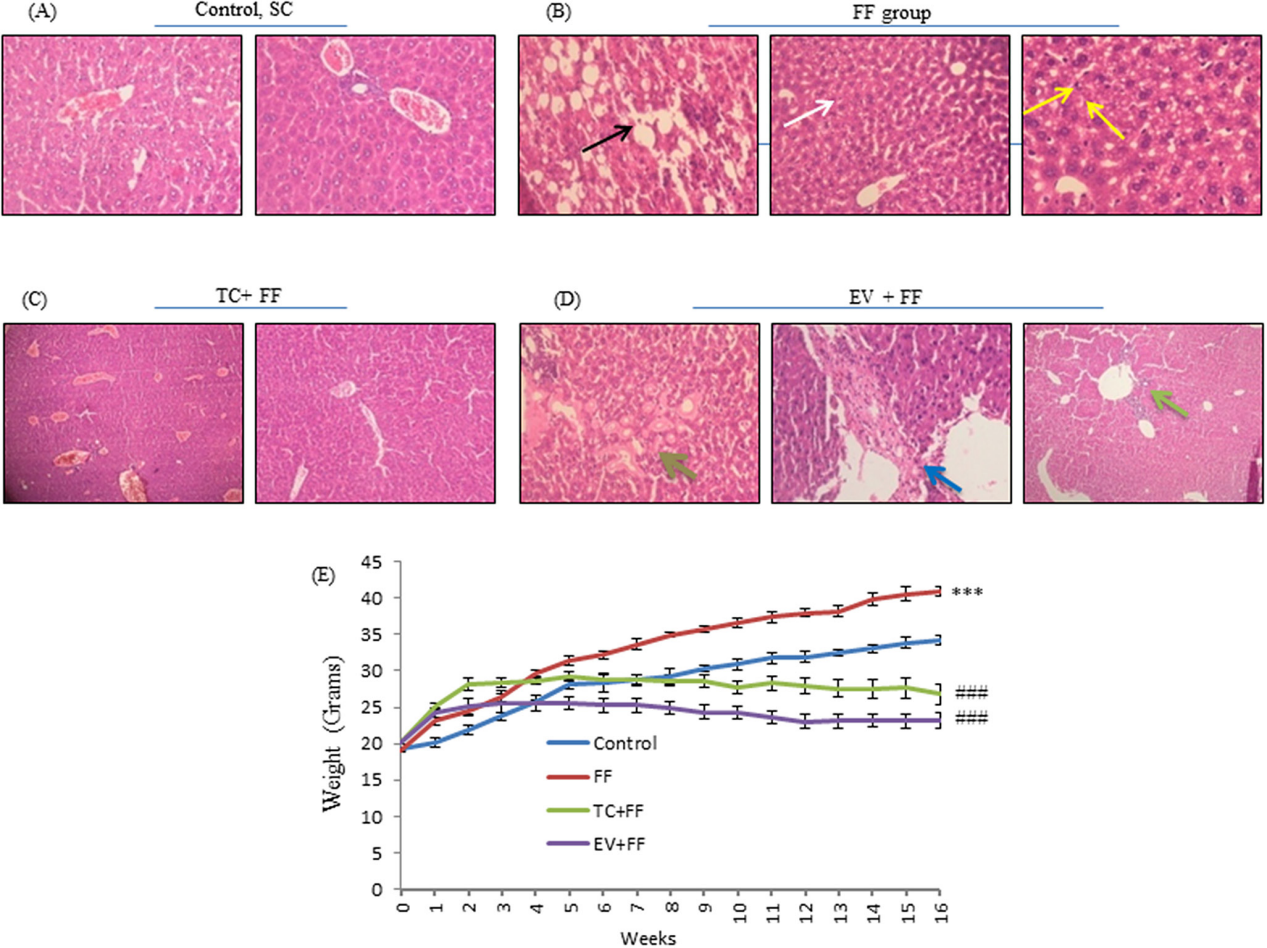
FF diet induced, steatosis, histological modifications, lipid droplet accumulation in C57BL/6J livers; and their prevention by TC and EV in co-administrative groups **A, B, C, D.** Representative photomicrographs (100 X) of H&E-stained liver sections of mice, after completion of study is shown. Analysis was done by expert histo-pathologist, who was blinded to the study. (A) Images correspond to mice reared on Standard Chow, show normal lobular architecture, with no signs of anomaly. (B) Images belong to mice reared with FF alone [FF group] shows micro and macrovesicular steatosis (black and white arrows), and activation of kuppfer cells (yellow arrow), suggesting an initiated fibrotic reaction but not complete fibrosis. (C) Images of TC group in which mice were fed with 1mg/kg of TC, thrice a week over a period of 120 days along with FF, show little or no sign of steatosis, improved liver histology, normal lobular architecture and normal parenchyma. (D) Images belonging to EV treated mice (1mg/kg, thrice a week) along with FF, also show no signs of steatosis, but inflammation and fibrotic reactions were observed in EV treated liver sections, suggesting wide portal tracks (dark green arrow) with acute and chronic inflammations (light green arrow) along with presence of amyloids (blue arrow), overall suggesting a fibrotic reaction in absence of significant deposition of lipids. **E.** Ogive graph shows weight gain pattern, over each week, by 4 groups, ^***^ shows significant weight gain by FF mice, FF versus SC, p<0.0001 and ^###^ shows significant decline in weight gain in TC & EV mice, TC versus FF, EV versus FF, p<0.0001. FF diet induced significant weight gain, shown by red line, as compared to Control group (blue). TC (green) and EV (violet) treatment prevented the weight gain in their respective groups, as compared to FF group.

The average weekly weight of mice in FF group increased till the end of the study and was 20% higher as compared to mice in SC group. Weight increased in TC group up to 5th week of the study while EV group showed weight increase up to the 3rd week only. However, at end of the study the average weight of both the groups was lowered by 34% in TC group and 44% in EV group as compared to FF fed mice (Figure 2B). Along with body weight, average liver weight of mice in FF group (1.67±0.15g) was also significantly higher than SC group (1.08±0.06g), (p<0.01); however, tacrolimus and everolimus prevented the increase in liver weights in TC and EV groups (also fed with FF diet), Table 1. Fasting serum levels of triglyceride, cholesterol, glucose and insulin were significantly high in FF group as compared to SC group. Tacrolimus and everolimus administration improved the levels of these blood parameters in FF fed mice (group TC and group EV). Homeostasis Model Assessment (HOMA)-IR [24] was 5.03 for FF group (p< 0.01) while only 1.88 for SC group, Table 1. Low adiponectin (ADP) level has a positive association with dyslipidemia [25], in our study the serum ADP levels in FF group were significantly reduced as compared to SC group (p<0.01), although TC & EV treatment improved the ADP levels, but this was not significant, (Table 1). FF fed mice developed the characteristics of MS. Administration of TC and EV in FF fed mice prevented the development of these features.

**Table 1:** FF induced metabolic derangements, weight gain in C57BL/6J mice; Tacrolimus and Everolimus prevented FF induced weight gain, and prevented altered metabolic profile in C57BL/6J

**Parameters**	Control, SC	FF	TC + FF	EV + FF
**Body Weight (gm)**	34.1 ± 1.6	40.9 ± 1.1^***^	26.7 ± 4.7^###^	23.2 ± 3.3^###^
**Liver Weight (gm)**	1.0 ± 0.0	1.7 ± 0.1^***^	1.0 ± 0.0^###^	1.0 ± 0.1^###^
**Liver (% of Body Weight)**	3.2 ± 0.0	4.4 ± 0.1^***^	3.8 ± 0.0^###^	4.2 ± 0.0^#^
**Serum cholesterol (mg/dl)**	121.6 ± 13.7	162.6 ± 25.4^*^	105.7 ± 2^##^	68.7 ± 15.9^###^
**Serum triglycerides (mg/dl)**	68.5 ± 12.4	91.0 ± 13.9^*^	67.5 ± 6.8^#^	72.4 ± 11.4
**Serum glucose (mg/dl)**	163.9 ± 37.3	291.7 ± 75.4^***^	103.5 ± 24.1^###^	69.5 ± 16.3^###^
**Serum insulin (ng/ml)**	2.1 ± 0.4	5.1 ± 0.5^***^	1.4 ± 0.1^###^	1.3 ± 0.0^###^
**Serum ADP (ng/ml)**	2.6 ± 0.3	1.8 ± 0.1^*^	2.1 ± 0.5	2 ± 0.4
**HOMA-IR**	1.88 ± 0.1	5.0 ± 0.1^***^	1.1 ± 0.1^###^	1.0 ± 0.0^###^
**Hepatic triglycerides (mg/mg protein)**	1.5 ± 0.0	19.0 ± 10.0^***^	1.7 ± 0.2^###^	2 ± 0.6^###^
**Hepatic cholesterol (mg/mg protein)**	0.69 ± 0.2	1.9 ± 0.7^**^	0.9 ± 0.1^#^	0.7 ± 0.1^##^

Values are represented as mean ± SD. ^*^ indicates p≤0.05, ^**^ indicates p≤0.01, ^***^ indicates p≤0.0001, FF versus SC. ^#^ indicates p≤ 0.05, ^##^ indicates p≤ 0.001, ^###^ indicates p≤0.0001, TC verses FF and EV verses FF.

FF induced significant weight gain, liver weights (LWs), liver to body weight ratios (LBWRs) (^***^ indicates p<0.0001: FF versus SC) were increased in C57BL/6J mice over period of 120 days. FF induced dyslipidemia, and deranged the metabolic profile indicated by high serum levels of cholesterol, triglycerides, glucose, insulin, and low levels of serum adiponectin (ADP), (^*^indicates p<0.05, ^**^ indicates p<0.01, ^***^ indicates p<0.0001: FF versus SC). With high serum levels of glucose and insulin, homeostatic model assessment of insulin resistance (HOMA-IR) was calculated, and it was significantly higher in FF (^***^ indicates p<0.0001: FF versus SC). FF also led to high triglycerides and cholesterol content in livers (^**^ indicates p<0.01, ^***^ indicates p<0.001: FF versus SC) of FF group. Tacrolimus and Everolimus administration for 120 days prevented the insulin resistance, dyslipidemia as shown with serum levels of triglycerides, cholesterol, insulin, and glucose. Triglycerides and cholesterol contents in livers of TC and EC groups were also reduced. (^#^ indicates p<0.05, ^##^ indicates p<0.01, ^###^ indicates p<0.001: TC vs FF, EV vs FF).

### Tacrolimus and everolimus treatment prevented lipid accumulation by reducing hepatic de-novo lipogenesis in C57BL/6J mice and immortalized mouse hepatocytes

**Figure 3:**
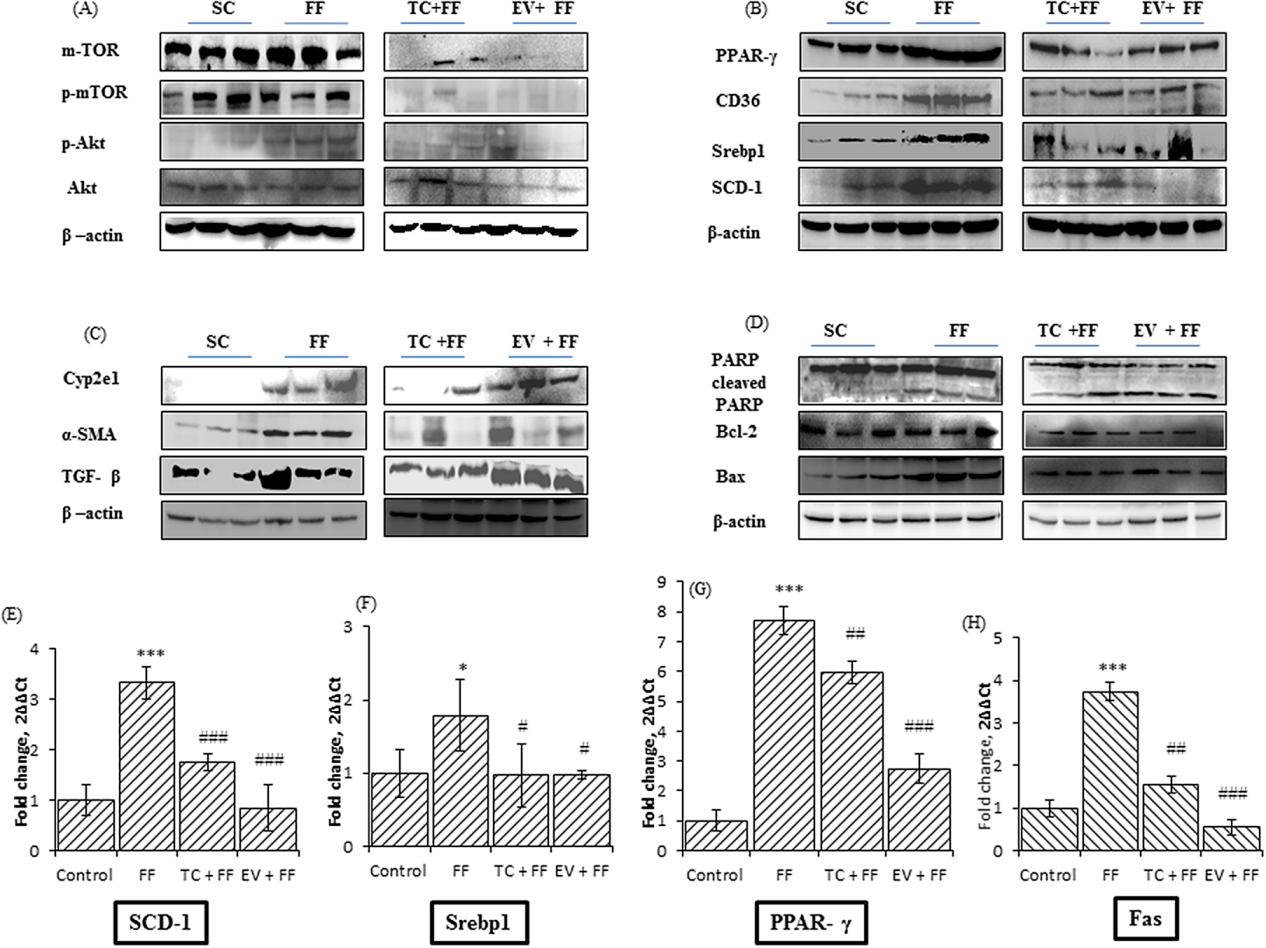
FF diet mediated protein and gene expressions changes in lipogenic and lipotoxic markers of hepatic tissues of C57BL/6J mice; Effect of 120 days administration of Tacrolimus and Everolimus along with FF diet on expressions of these markers **A, B, C, D.** are the representative blots of whole liver tissues lysates for specified antibody. Livers of three mice of each group were randomly selected and lysates were prepared, were resolved on SDS-PAGE, transferred to PVDF membrane and immunoblotted with the specified antibodies, and followed with detection of bands by enhanced chemiluminescence. The blots were quantified by Image Lab software (Bio-Rad) and normalized to β-actin. The blots for each antibody were run thrice at least and images here, are representative. (A) Images shown are the representative blots of proteins (m-TOR, p-mTOR, p-Akt, Akt) involved in insulin signaling, and further regulating de-novo lipogenesis. Fast food enhanced the activity of m-TOR, shown by increased expression, along with increased phosphorylation of m-TOR, which further enhanced the phosphorylation of Akt. TC and EV along with FF diet in their respective group, prevented the up-regulation of m-TOR, and thereby of p-Akt, also, hence preventing the hepatic insulin resistance. (B) Images shown are the representative blots of PPAR-y, CD-36, SREBP1, and SCD-1., all which are involved in hepatic lipid metabolism, i.e. denovo-lipogenesis. FF diet increased the protein expressions of m-TOR regulated transcription factors PPAR-y and SREBP1, which further enahanced the lipogenesis, by increasing the activity of SCD-1, and fatty acid flux associated protein CD36. TC and EV co-treatment inhibited the increased de-novolipogenesis, by reducing the protein expressions of PPAR-y, SREBP-1, CD36, and SCD-1. (C&D) Images shown are the blots of proteins involved in cell death, inflammation and fibrotic changes. 120 days administration of FF diet induced lipotoxicity and inflammation in livers of FF group. The hepatic expression of Bcl2-Bax was low (FF versus SC) in FF group while cleaved PARP was increased (FF versus SC) indicating FF induced cell death in livers. FF up-regulated the proteins associated with inflammation and activation of HSCs. The expressions of α-SMA, Cyp2E1 and TGF-β were significantly increased (FF versus SC). TC treatment in C57BL/6J prevented the FF induced lipo-toxicity, cell death. TC group livers have increased bcl2-bax ratio and reduced cleaved PARP (FF versus TC group). Tacrolimus treatment also prevented the FF induced inflammation and activation of HSCs, as α-SMA, TGF-β were reduced in TC group livers (FF versus TC group), but there was no change in expression of Cyp2E1. Chronic administration of EV showed toxic manifestations in livers of EV group, despite the minimal steatosis. Cyp2E1 was over-expressed in EV group while the expressions of α-SMA, TGF-β, cleaved PARP, bcl2-bax were not improved in livers of EV group, compared with FF group. **E, F, G,** and **H.** Images are bar graph of indicated hepatic gene expressions, performed by rt-PCR. rna was extracted, from the randomly selected 3 liver tissues of each group, transcribed to cDNA, quantified and pooled for 20uL reaction run on Roche, Light Cycler (LC-96) using 1X SYBR Green, forward and reverse primer, and rt-pcr grade water, and taking GAPDH as housekeeping gene and reactions were performed in triplicates. FF diet induced the gene expression of Scd-1 by 3 fold as compared with SC group. It also increased the expression of PPAR-y by 7 fold and of Fas by 3.8 fold as compared with SC group (^***^ indicates p<0.001, FF versus SC group). TC and EV prevented the up-regulation of these lipogenic genes, significantly. TC and EV prevented FF diet induced up-regulation of Scd-1 (^###^ indicates p<0.001, TC versus FF group, EV versus FF group), Srebp1 (# indicates p<0.05, TC versus FF group, EV versus FF group), PPAR-y and Fas (^##^ indicates p<0.01, TC versus FF group, ^###^ indicates p<0.001, EV versus FF group).

**Figure 4:**
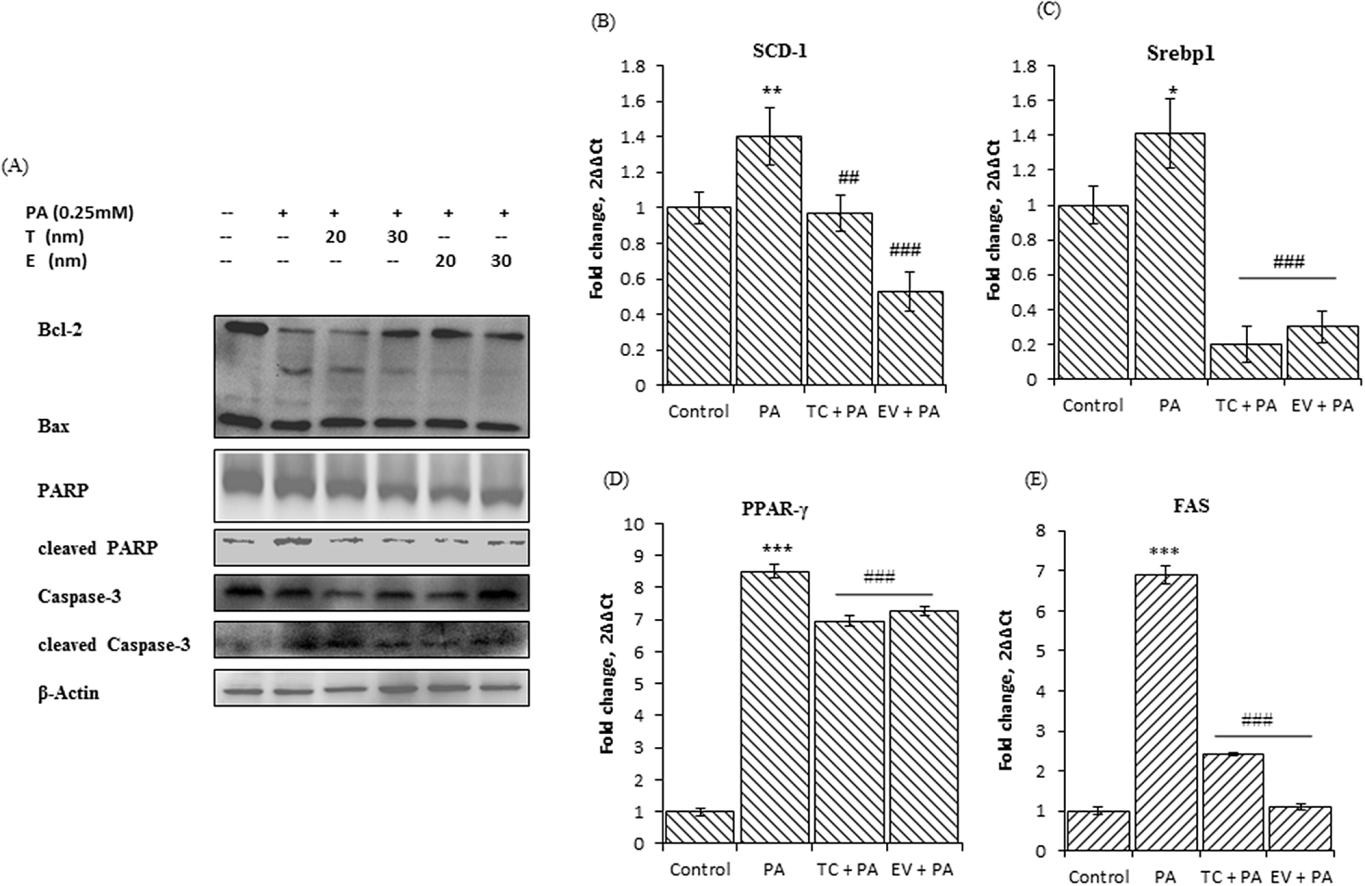
Palmitate induced up-regulation of lipogenic genes, and induced lipotoxicity, suggested by changes in expression of apoptotic markers in AML-12 cells Effect of Tacrolimus and Everolimus on these events mediated by palmitate in AML-12 cells. **A.** Image shows western blots for indicated proteins of whole cells lysate of AML-12 treated in a same manner as described earlier. Bands were detected by chemiluminescence, quantified by Image-lab (Bio-Rad) software and normalized to β-actin. Blots were run thrice, for each antibody and representative blots are shown in this figure. Palmitate treated cells have low bcl2-bax ratio, and increased cleaved PARP & cleaved caspase-3 indicating cell death as consequent of palmitate induced lipotoxicity. TC and EV pre-treatment prevented the palmitate induced lipotoxic events in AML-12 cells. Bcl2-bax ratio was increased at Tacrolimus 30nM and at Everolimus 20nM & 30nM. Expressions of cleaved PARP & cleaved caspase-3 were reduced significantly in Tacrolimus and Everolimus treated AML-12 cells. **B, C, D, E.** Images are the fold changes in expression of indicated genes (run on rt-pcr, as described earlier) as a result of exposure to palmitate (0.25mM) alone and/or with TC & EV. Palmitate (0.25mM) induced the significant up-regulation of lipogenic genes Scd-1 (^**^ indicates p<0.01, Palmitate versus Control, un-exposed cells). Srebp1 (^*^ indicates p<0.05, Palmitate versus Control), PPAR-y and Fas (^***^ indicates p<0.001, Palmitate versus Control). TC and EV treatment along with palmitate 0.25mM) significantly prevented the up-regulation of these genes. (^##^ indicates p<0.01, ^###^ indicates p<0.001, as represented, for either TC versus FF, or EV versus FF).

**Figure 5:**
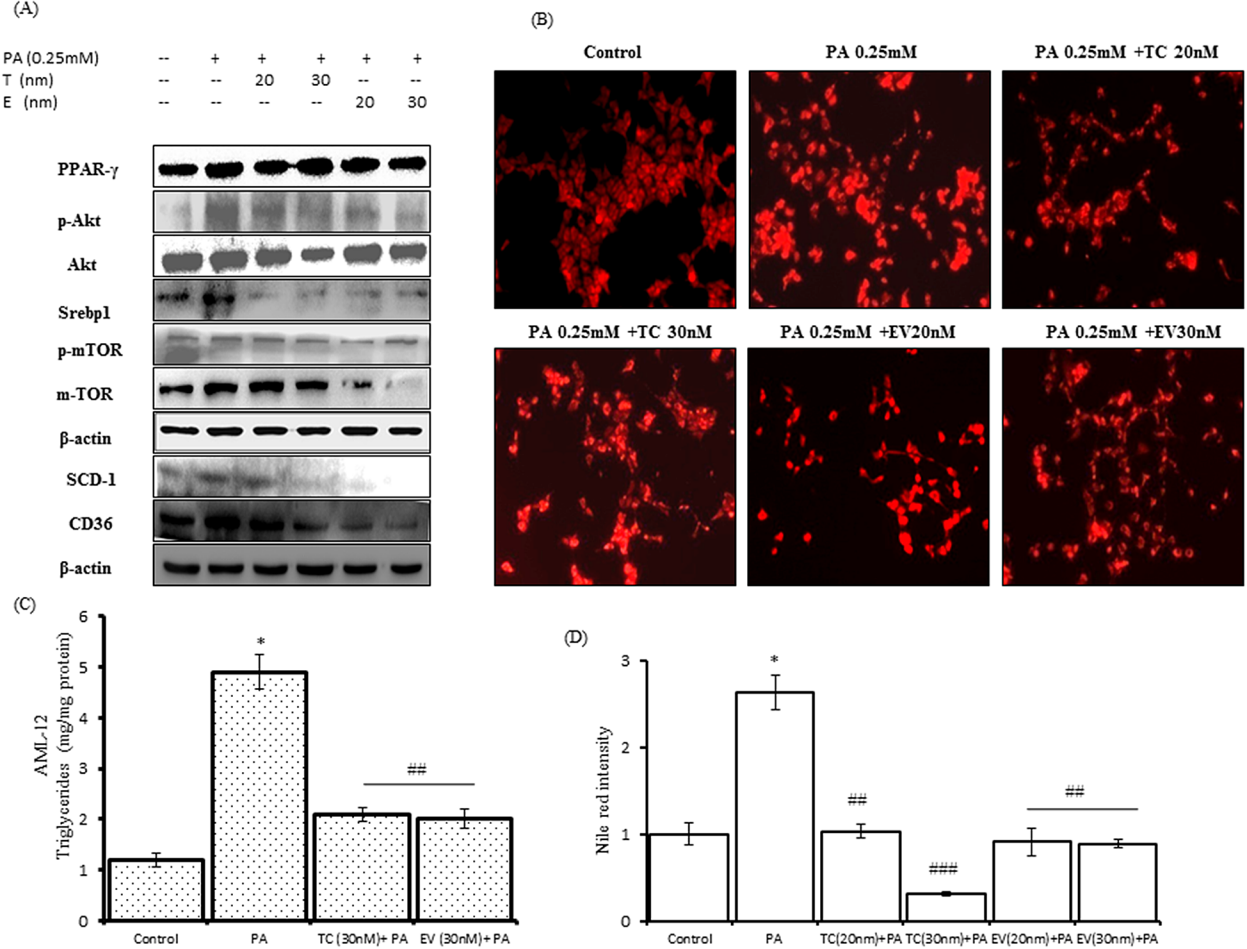
Effect of palmitic acid on protein expressions of key lipogenic players in AML-12 hepatocytes, LD accumulation and triglycerides content in AML-12 hepatocytes and its prevention by co-treatment with Tacrolimus and Everolimus AML-12 cells were exposed to palmitic acid (0.25mM) and pre-treated with TC (20nM and 30nm), EV (20nM and 30nm) 4 hours before exposure to palmitic acid for 24 hours. **A.** Western blots of whole cells lysates for indicated proteins, which were resolved on SDS-PAGE, transferred to PVDF membrane, immunoblotted to specific antibodies. Bands were detected by chemiluminescence, quantified by Image-lab (Bio-Rad) software and normalized to β-actin. Blots were run thrice, for each antibody and representative blots are shown in this figure. Tacrolimus @ 20nM & 30nM reduced the expression of PPAR-γ, p-AKT and SCD-1. Tacrolimus 30nM also reduced the expressions of p-mTOR, m-TOR and CD36; however the reductions of these proteins with TC 20nM were not significant. EV treatment in AML-12 cells also prevented the up-regulation of palmitate induced PPAR-γ, m-TOR, p-AKT, SCD-1. EV treatment reduced the expressions of CD36 while EV 20nM reduced the expression of p-mTOR. TC and EV @ 20nM & 30nM significantly (p<0.05) reduced expression of SREBP1. **B.** Shows images for nile red stained AML-12 cells, which were palmitate exposed alone and/or pre-treated with TC and EV for 20nM and 30nM 4 hours before palmitate exposure, total of 24 hours. **C.** Bar graph show triglyceride content, in AML-12 cells, after 24 hours of exposure with palmitate @ 0.25mM and/or pre-treatment with TC and EV. Palmitate exposed cells have 5 fold higher content of triglycerides as compared to control untreated cells (^*^ p<0.001), while TC and EV treated AMl-12 cells, had significantly reduced triglyceride content (^##^ indicate p<0.001). **D.** Fold change intensity of fluorescence measured by Image J software (NIH, USA). The palmitate exposed cells had increased fluorescence (^**^ indicates p<0.01: Palmitate versus Control AML-12 cells, untreated and unexposed). TC and EV at 20nM and 30nM concentrations prevented the palmitate accumulation as visible from decreased fluorescence in their respective images (^##^ indicates p<0.01, ^###^ indicates p<0.001: Palmitate versus TC 20nM & 30nM, Palmitate versus EV 20nM & 30nM).

Lipid accumulation in hepatocytes and hepatic triglyceride content are the main mediators of fatty liver disease. We analyzed the lipid accumulation in AML-12 hepatocytes by nile red staining, while H&E staining of liver sections obtained from C57BL/6J mice revealed the extent hepatic lipid accumulation in mice. H&E stained images of FF group showed excessive lipid accumulation with macro and micro-vesicular steatosis (Black and White arrows), as compared to images of SC group. TC and EV administration prevented the lipid accumulation and there were very little signs of steatosis in H&E images of TC and EV groups. In palmitate-exposed AML-12 cells, lipid accumulation was increased, shown by enhanced fluorescence (Figure 3B, PA 0.25mM) as compared to untreated cells (Figure 3B, Control cells), and it was 2.4 fold more in palmitate treated cells only as compared to control cells. TC & EV treatment prevented the lipid accumulation in AML-12 cells induced by palmitate exposure (Figure 4B, 4C). Lipid droplet (LD) accumulation has been associated with high triglyceride content in liver tissues/hepatocytes [26]. FF fed mice showed 18-fold higher triglyceride content in their livers as compared to SC group (Table 1). AML 12 cells treated with palmitate also showed 5-fold increase in triglyceride content as compared to untreated cells (Figure 5C). The triglyceride content in liver tissues of TC & EV administered groups reduced significantly (p<0.001) which was comparable with triglyceride content of SC group livers, while TC and EV treatment in AML-12 hepatocytes also reduced the triglyceride content significantly (p<0.001) as compared to palmitate-exposed AML-12 cells. Further, we analyzed the effect of TC and EV on protein expressions and m-RNA levels of major proteins involved in lipogenesis. Protein expressions of m-TOR & p-mTOR were up-regulated in livers of FF group and AML-12 cells exposed to palmitate (0.25mM) for 24 hours. The expression of m-TOR was increased by 5 fold (p<0.001) in livers of FF group while p-mTOR was increased by 1.5 fold. m-TOR hyperactivity was associated with over-expression of p-Akt by 1.65 fold in livers of FF group as compared to livers of SC group (Figure 3A). Similarly, palmitate-exposed AML-12 cells, mTOR and p-mTOR levels increased by 1.7 and 1.4 fold respectively whereas p-Akt levels increased by 2.2 fold as compared to untreated control cells (Figure 5A).

The expression of m-TOR mediated transcriptional factors such as SREBP1 and PPAR-γ was also increased in livers of FF group. This up-regulation of SREBP1 & PPAR-γ in FF fed mice was associated with 3.4-fold increase in cluster of differentiation 36 (CD36) expression and 1.45-fold increase in SCD-1 levels as compared to SC group (Figure 5B). Similarly, palmitate-exposed AML12 cells also showed increased expression of PPAR-γ (1.8 fold), SREBP1 (1.7 fold) and SCD-1 (2.3 fold), Figure 4A. We also analyzed the hepatic m-RNA levels of main players involved in de-novo lipogenesis. The hepatic m-RNA levels of transcriptional factors PPAR-γ, was 8 fold higher in FF group, SREBP1 mRNA levels were 1.8 fold increased, but insignificant, hepatic mRNA levels of CD36 and SCD-1 also increased by 3.7 and 3.3 fold respectively. The mRNA expression of fatty acid synthase (FAS) was also increased by 3.7 fold as compared to SC group (Figure 3E, 3F, 3G, 3H). Palmitate treatment also up-regulated the m-RNA levels of these proteins. m-RNA levels of PPAR-γ and FAS were increased by 8 and 6.9 fold respectively (p<0.001), while m-RNA levels of SREBP1 & SCD-1 were significantly increased by 1.4 & 1.5 fold respectively as compared to non-treated control cells (Figure 4B, 4C, 4D, 4E).

TC and EV inhibited the denovo-lipogenesis in C57BL/6J livers and in AML-12 hepatocytes. The protein expression of m-TOR was totally inhibited in EV group and phosphorylated m-TOR was significantly reduced by 73% (p<0.0001) as compared to FF group. Similarly, in TC group, the levels of m-TOR & p-mTOR were significantly reduced by 67% (p<0.0001) & 41% (p<0.001) respectively. Transcriptional factor PPAR-γ was reduced by 72 % (p<0.0001) in both TC & EV groups, while that of SREBP1 was reduced by 40% (p<0.001) in TC group & 50% (p<0.001) in EV group. The hepatic protein expression of SCD-1 in treated groups was reduced by 25% (p<0.05), compared with FF group, and on the other side expression of CD36 was also down by 33% (p<0.01). TC and EV treatment also affected the hepatic m-RNA levels of ChREBP1, CD36, and SCD-1 which were significantly reduced as compared with m-RNA levels analyzed in mice of FF groups. Following the similar trend, the hepatic m-RNA levels of SREBP1, PPAR-γ were also significantly reduced by TC and EV treatment (Figure 3E, 3F, 3G and 3H).

TC and EV treatment in palmitate-exposed AML-12 hepatocytes also showed similar effect on the protein and m-RNA expressions of molecular players that were studied in C57BL/6J. The protein expressions of PPAR-γ, SREBP1, m-TOR, p-mTOR, p-Akt, SCD-1, CD36 were significantly reduced by TC and EV treatment. The m-RNA levels of these proteins were analyzed in AML-12 cells, which revealed that TC and EV significantly reduced the m-RNA levels of PPAR-γ (p<0.0001), FAS (p<0.0001), SREBP1 (p<0.0001) & SCD-1 in palmitate treated cells (Figure 4B, 4C, 4D, 4E). These results confirmed that increased de novo lipogenesis in FF fed mice and palmitate treated mouse hepatocytes is significantly reduced by TC and EV.

### FF induced steatohepatitis, and early features of fibrosis in C57BL/6J: characteristic of NASH

H&E staining of FF group livers showed evidence of inflammation (White arrow) and early signs of fibrosis including ballooning and activation of kupffer cells (Yellow arrow) (Figure 2B), while in SC group we observed normal lobular architecture with normal portal tract (Figure 2A). Apoptosis was also evident in livers of FF fed mice as confirmed by poly [ADP-ribose] polymerase 1 (PARP1) cleavage and decreased bcl2/bax ratio. The level of cleaved PARP was significantly higher (2.5 fold) while bcl2/bax ratio was only 0.6 in FF group when compared with SC group. Moreover, we studied expressions of key inflammatory and fibrotic protein markers in livers of mice, in which the Cytochrome P450 2E1 (CYP2E1) was found to be 1.8 times over-expressed in FF group. The expression of fibrotic markers i.e. alpha-smooth muscle actin (α-SMA) and transforming growth factor beta (TGF-β) was increased in FF group by 1.7 and 2 fold respectively (Figure 3C, 3D). These results indicate that 120 days feeding of FF diet induced inflammation and fibrosis as a feature of NASH.

### Everolimus showed potent anti-steatotic effect, but inflammation and fibrosis were evident

EV group livers showed no signs of lipid accumulation with minimal micro & macro-vesicular steatosis (Figure 2D), but the surprising finding was presence of inflammation and fibrosis. H&E staining showed, foci of acute and chronic inflammation (dark green arrow) with deposition of pink amorphous acellular material (blue arrow) in walls of blood vessels nourishing the tissue and enlarged portal tract with chronic inflammation (light green arrow). These events were associated with significant up-regulation of cleaved PARP, α-SMA, TGF-β with low bcl2-bax ratio, as compared with SC group (Figure 3C, 3D).

### Tacrolimus prevented steato-hepatitis, showed anti-fibrotic activity, and improved liver histology

H&E staining of TC group livers showed no signs of fatty liver and showed improved histopathology of liver as confirmed by normal parenchyma and normal lobular architecture (Figure 2C). Tacrolimus prevented the up-regulation of α-SMA & TGF-β as compared with EV group and FF group. TC treatment also attenuated the FF induced cell death, as expression of PARP1 was reduced by 48%, almost equivalent to SC group while the bcl2/bax ratio was up by 30% i.e. 0.9 as compared to FF group (Figure 3B, 3C).

### Tacrolimus & everolimus attenuated palmitic acid-induced lipotoxic events in AML-12 hepatocytes

To confirm the findings of our *in-vivo* study, we analyzed the lipotoxic events mediated by palmitic acid and the role of TC & EV in preventing those events in AML-12 hepatocytes. Twenty-four-hour exposure of palmitate (0.25mM) to AML-12 cells, led to significant decrease in cell viability (20%, p<0.05) (Supplementary Figure 1E), and subsequent up-regulation of PARP1 (2.5 fold) and of cleaved caspase-3 (1.9 fold), (Figure 4A). The bcl2/bax ratio was also reduced by the palmitic acid treatment (Figure 4A). EV pre-treated AML-12 hepatocytes showed resistance to lipotoxicity. Protein expressions of cleaved PARP and cleaved caspase-3 were significantly reduced by 60% (p<0.001) & 22% (p<0.05) respectively, along with significant up-regulation, (p<0.05) of bcl2-bax ratio, as compare to palmitate exposed cells. TC also complemented its anti-lipotoxic property in AML-12 cells.

## DISCUSSION

In this study, our aim was to analyze the long term (16 weeks) preventive efficacy of Tacrolimus and Everolimus administration in an experimental mouse model of NAFLD and subsequently NASH, induced by FF. The principal findings of our study show the anti-steatotic potential of both drugs, by targeting key transcriptional factors involved in lipogenesis. However, EV treatment induced inflammatory and fibrotic responses despite antisteatotic effect.

Experimental NASH was established by rearing genetically unaltered C57Bl/6J mice on FF diet for 16 weeks. Denovo-lipogenesis and lipotoxicity induced by FF diet and the therapeutic efficacy of TC and EV in preventing these events was studied. The key features related to lipogenesis and lipotoxicity, induced by FF diet in mouse model were also confirmed in palmitate overloaded AML-12 mouse hepatocytes. FF diet developed steatosis (first hit), which has also been observed in conventional NAFLD models, utilizing small animals. In such models, either high fat or high fructose is usually administered and these models have been reported to cause NAFLD either independently or in combination [27-30]. However, FF diet model, comprises of high cholesterol, high fat diet along with fructose. This model produced the biochemical and histological NASH conditions that closely recapitulates the human condition [8]. We have previously investigated the chronology of these phenomena in FF diet-induced animal model of NASH. In this model, male C57Bl/6 mice were randomly assigned to a fast food (FF) or a standard chow (SC) diet and reared up to 16 weeks. Fructose was provided in the drinking water. Serum insulin and cholesterol levels were multiphasic in FF mice rising significantly (p<0.001) by week 1, normalizing to baseline levels by 4 weeks and thereafter increasing 2-4 fold by 16 weeks. Insulin resistance (measured by HOMA IR) was observed by 4 weeks (p<0.05) and was persistently high (FF=63-77 vs SC=18-23, p<0.001) beyond 8 weeks. NASH was apparent histologically by 16 weeks [31]. The timeframe of 16 weeks was sufficient to reproduce most significant stages of fatty liver disease, from initiation of steatosis to hepatic inflammation, leading to hepatocyte ballooning and fibrosis. Therefore, we evaluated the efficacy of TC and EV in FF fed mice for 16 weeks diet model.

By 16 weeks, C57BL/6J mice reared on FF diet potentially induced features of MS, with elevated levels of triglyceride, cholesterol, and insulin resistance (elevated serum levels of glucose and insulin and low serum levels of ADP). NAFLD was confirmed histologically in FF treated mice by increased presence of macro & micro-vesicular lipid droplets and biochemically by increased hepatic triglyceride content. Hepatic expressions of m-TOR and p-mTOR were up-regulated in mice reared on FF diet alone. Likewise, protein expressions analysis of m-TOR and p-mTOR were up-regulated in palmitate (0.25mM, for 24 hours) exposed AML-12 hepatocytes. This confirms a crucial role for m-TOR activation in lipogenesis leading to induction of NAFLD. We also found the activation of Akt, in livers of mice reared on FF diet and in palmitate exposed AML-12 cells, as indicated by increased p-Akt expression. Activation of m-TOR and Akt-mediated by palmitate or FF has been shown to be involved in development of insulin resistance in cells or tissues and in hepatocytes and skeletal muscles [32]. Excess supply of fatty acids to hepatocytes has been shown to be involved in stimulation of m-TOR and its upstream S6 kinase, which further co-relates with the system specific insulin resistance [33]. In our study, we confirm insulin resistance, with de-arranged serum levels of glucose, insulin and ADP, and along this hepatic activation of m-TOR, p-mTOR and Akt was also involved.

**Figure 6:**
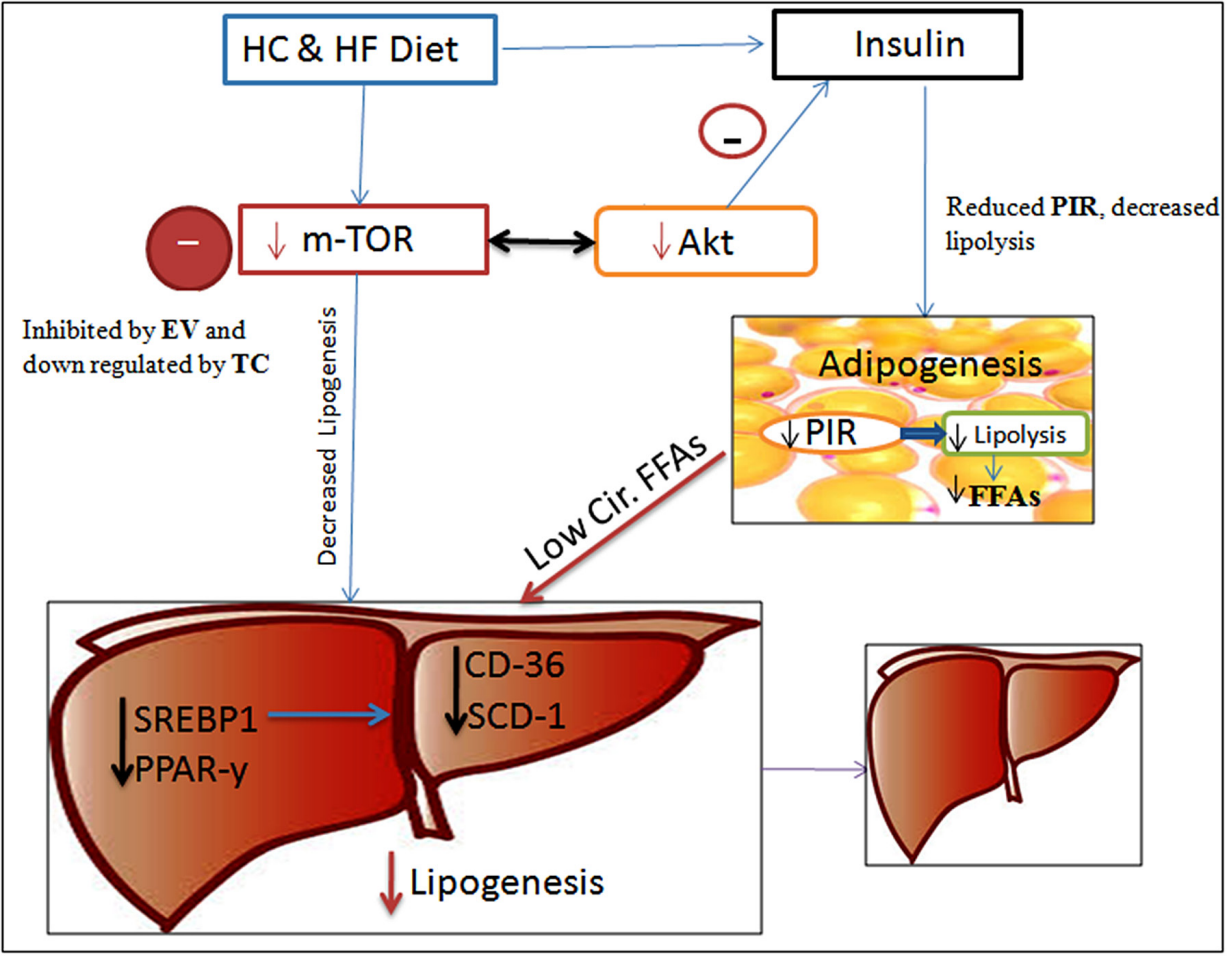
Schematic Diagram showing the possible effects of TC and EV administration along with FF diet m-TOR up-regulation by FF diet was prevented by TC and EV administration, which led to decreased hepatic lipogenesis, because of down-regulation of associated hepatic lipogenic players viz. SREBP1, PPAR-y and SCD-1. EV and TC also modulated m-TOR-Akt-insulin signaling and prevented insulin burst, counteracting PIR and HIR. Since PIR could not develop, lipolysis was reduced resulting in to low circulatory FFAs towards liver/hepatocytes which further prevented enhanced denovo-lipogenesis. Hence lipid accumulation was minimal in TC and EV treated livers of C57BL/6J mice fed on FF diet. Abbreviations: FFA-Free fatty acids, HC & HF – High carbohydrate and high fat, HIR – Hepatic insulin resistance. PIR-Peripheral insulin resistance.

TC and EV treatment improved the consequences of FF diet feeding in C57BL/6J (Figure 6), and exposure of palmitate in AML-12 cells. EV being a potent m-TOR inhibitor [32], prevented the LD accumulation, reduced hepatic triglyceride content, and improved insulin resistance. We also found improved insulin sensitivity (Supplementary Figure 1A) and normalized metabolic profile in EV treated mice reared on FF diet. However, glucose intolerance, and impaired insulin resistance with administration of Rapamycin (m-TOR inhibitor) has been reported [12, 14, 33], whereas sirolimus (Rapamycin) has also been reported to increase insulin mediated glucose uptake [34]. We found significant decline of weight gain in mice of EV group, similar to the other studies in which m-TOR inhibition with Rapamycin prevented weight gain in mice fed over high-fat diet [34-37]. We administered EV thrice a week by oral gavage and achieved therapeutic outcomes in 16 weeks contrast to the other study, where therapeutic outcome was poor with oral administration of Rapamycin [38]. EV treatment for 16 weeks completely inhibited the expression of m-TOR in liver tissues of EV group. m-TOR levels were also inhibited in AML-12 cells, which were pre-treated with EV before exposing to palmitate. Chronic m-TOR inhibition by EV in our study improved the metabolic outcome in C57BL/6J mice that were fed FF diet, contrast to others, where it had no positive effect or even a worsening effect [12, 33, 37]. There are a few studies that show m-TOR inhibition leads to improved metabolic alterations [35, 36] however they have not employed Everolimus for m-TOR inhibition. Therefore, the present study through a spot light at a new dimension for everolimus related m-TOR inhibition to improve metabolic outcome. However more elaborate and long-term studies in varied experimental models are needed to confirm this for its suitability in clinical studies, as our results indicate that EV treatment inspite of improving metabolic parameters, induced inflammatory and fibrotic responses.

Tacrolimus (TC), a calcineurin inhibitor, has been reported to have a controversial role in development and prevention of steatosis, or new onset of diabetes mellitus in clinical and *in-vivo* settings. Tacrolimus treatment inhibited FF diet induced lipid accumulation in liver tissues of mice treated with TC (TC group) and in palmitate exposed AML-12 hepatocytes. The novel phenomenon which we observed with TC treatment in mice and in palmitate exposed AML-12 cells was the down-regulation m-TOR and p-mTOR. Although, TC mediated down-regulation of m-TOR levels were not as potent as with EV treatment, but it was significant in both *In-vitro* (AML-12 cells) and *In-vivo* (TC group). Other features included improved metabolic profile and lipid-glucose biochemistry. Along with m-TOR, and p-mTOR, TC treatment also prevented the activation of Akt in liver tissues of TC group mice and in palmitate exposed AML-12 cells, which signifies the role of TC normalizing the insulin signaling resulting in improved insulin sensitivity, leading to decreased LD accumulation and reduced hepatic triglyceride content. This is contradictory to some clinical and animal model studies where the role of tacrolimus in new onset of diabetes mellitus, inducing steatosis in grafted liver and promoting post transplant diabetes mellitus has been suspected [15, 16, 17].

Transcriptional factors SREBP1 and PPAR-γ are mediated via m-TOR and their increased activity indicate enhanced lipogenesis which germane lipid droplet formation [39]. Both these transcriptional factors were over expressed in livers of FF diet fed mice (FF group) as evident from increased protein and m-RNA expressions of related genes. Hepatic SREBP1 and PPAR-γ activation stimulate lipogenesis by enhancing the activity of adipogenic genes viz. fatty acid synthase (FAS), SCD-1 & CD36 that are involved in lipid partitioning, poly-unsaturated fatty acids (PUFA) synthesis and influx of fatty acids in hepatocytes [39], and these were elevated in liver tissues of FF diet fed mice. We confirmed these events in AML-12 cells, where palmitate exposure enhanced the mRNA/protein levels of FAS, SCD-1 & CD36.

In liver tissues of TC & EV treated mice reared on FF diet, down-regulation of SREBP1, PPAR-γ diminished the activities of FAS and SCD-1, while there was also decreased influx and transport of fatty acids as TC & EV treatments prevented CD36 up-regulation in mice and in AML-12 cells exposed to palmitate (Figure 6). Apart, from denovo-lipogenesis, and diet, liver receives major fatty acid flux from circulation and circulatory fatty acids are derived from peripheral lipolysis, which is usually hastened in insulin resistant state [40]. TC and EV treatments to FF diet fed mice decreased the insulin resistance and improved the insulin sensitivity, which led to reduced circulatory fatty acid load resulting in low triglyceride content and LD accumulation.

In FF diet fed mice (FF group), steatosis with advanced steatohepatitis and initiation of fibrotic reactions (histological features of NASH) were markedly apparent at 16 weeks of FF diet consumption. This group also had highly significant apoptosis and cell death, which was confirmed from increased hepatic expression of cleaved PARP and low bcl2-bax ratio. However, the features of inflammation, apoptosis and fibrotic reaction were also present in liver tissues of EV treated mice fed on FF. This group also exhibited increased apoptosis, with low bcl2-bax ratio and increased expression of cleaved PARP, and α-SMA [a hallmark of hepatic stellate cells (HSCs) activation], thus resulting in promotion of extracellular matrix (ECM). FF diet group (FF group) and EV treated, FF diet fed group (EV group) also presented with increased levels of CYP2E1, which is positively correlated with inflammation and acute liver injury [41]. Enhanced collagen synthesis was expected in both these groups as an increased expression of TGF-β was found, which also plays a key role in inflammation and deposition of extracellular matrix [42]. All these molecular inferences related to inflammation and fibrotic reactions in both groups were confirmed by H&E staining of liver tissues as well. The toxic manifestation in liver tissues of EV treated mice fed on FF diet (EV group) may be associated with two reasons: a) Chronic inhibition of SCD-1, b) Autophagy-related fibrogenesis. SCD-1 is supposed to play a protective role in liver, and scd-1 null mice have shown increased hepatic apoptosis [43]. Previously [24], we have shown that, HepG2 cells were resistant to lipotoxicity, owing to increased sensitivity towards SCD-1. In the present study we performed lipotoxicity analysis in AML-12 cells exposed to palmitate (0.25mM), one hour pre-incubated with SCD-1 inhibitor (MF-438, Calbiochem) where we found enhanced lipotoxicity (40-42% cell death) as compared to cells exposed to palmitate only (20-21% cell death) (Supplementary Figure 1C). SCD-1 was inhibited completely (Figure 2B) by EV treatment in C57BL/6J mice fed on FF diet and inhibition was sustained and chronic, which could trigger apoptosis. However, EV treatment showed protection against lipotoxicity induced by palmitate exposure, with improvement of anti-apoptotic markers, even though SCD-1 inhibition was also significant in AML-12 cells. This suggests that sustained and chronic inhibition of SCD-1 with EV treatment, in *in-vivo* conditions, sensitized liver cells to apoptosis and cell death. Secondly, everolimus is known an autophagy inducer, and autophagy in HSCs promotes fibrogenesis [44] which could be another plausible reason behind fibrotic reaction in liver tissues of EV treated mice fed on FF diet (EV group), which has occurred as independent of steatosis, suggesting the pro-fibrogenic behavior of EV, which is quite contradictory to other studies in which everolimus and its sister drug rapamycin (sirolimus) proved to attenuate fibrosis in different organ types [13]. However, studies have highlighted both success and failure stories for rapamycin and everolimus in treating established liver fibrosis [45, 46]. In Mdr2 −/− mice, m-TOR inhibitors lacked efficacy, while everolimus treatment inhibited activated HSCs in *In-vitro* and *In-vivo* models [47], this clearly suggest that m-TOR inhibitors as anti-fibrotic act differently in varying etio-pathogenesis and *In-vivo* models, so further studies are required to make this scenario much more conclusive.

As discussed earlier, tacrolimus proved to be a potent anti-steatotic agent, and also prevented steatohepatitis. TC treatment to mice fed on FF diet (TC group) prevented the lipotoxicity in C57BL/6J liver tissues which were further confirmed by improved bcl2-bax ratio, reduced expression of cleaved PARP. These manifestations were confirmed in AML-12 cells exposed to palmitate. TC treated cells, showed reduced cleaved caspase-3 expression induced by palmitate exposure as well. The protein expressions of α-SMA, Cyp2E1, and TGF-β were reduced in TC treated mice fed on FF diet (TC group), which suggest that tacrolimus prevented FF diet induced inflammation, and ECM synthesis. The histopathological analysis of liver sections of TC treated mice fed on FF diet showed reduced lipid accumulation (steatosis) with a normal histology. The role of tacrolimus as an anti-fibrotic nature is dicey, with respect to induction or prevention of fibrosis in different organs including liver [18, 19, 20, 21 and 48]. However, present study highlights the novel role of tacrolimus in preventing the fibrosis and liver injury induced due to consumption of fat, cholesterol and high fructose.

## CONCLUSION

In summary, this study suggests that tacrolimus and everolimus are potent anti-steatotic agent, and Tacrolimus also prevented steatohepatitis and fibrosis, which makes it a suitable candidate for further elaboration of its role in treating NAFLD/NASH. More studies on everolimus are warranted in different NAFLD models. Dosing titration, formulation and period of administration are certain variables that should be of prime concern for such studies.

## MATERIALS AND METHODS

### Animals and treatments

Four to six weeks old, genetically unaltered male C57BL/6J (16-18g weight) mice were procured and housed in institutional animal care facility. Mice were acclimated for one week prior to start of the experiment and study was approved by institutional animal ethical committee (IAEC). The fast food diet used in this study, has been previously described well in detail [8]. Mice were divided into four groups (n=8). Group 1 Standard Chow [SC], Group 2: FF diet, Group 3: FF + Tacrolimus [TC], Group 4: FF + Everolimus [EV group]. The drugs were administered orally @ 1mg/kg three times per week [14, 19], by preparing as aqueous suspension in 0.5% of DMSO, while SC and FF groups were also administered with blank solution (with 0.5% DMSO), during every dosing schedules. The study was scheduled for 120 days. All the mice were caged separately to encourage sedentary pattern [8], with conditions 24° ± 2°C, 12h light/dark cycle and had free access to respective diets for 16 weeks. Weekly weight pattern (Figure 1E) and food intake (Supplementary Figure 1F) were recorded, and mice were sacrificed by CO_2_ euthanasia. Blood was collected by cardiac puncture, and centrifuged; serum was stored at -80°C. Livers were harvested, snap frozen for further western blotting, rt-PCR or stored in 10% formalin for histopathology staining.

### Serum biochemistry

Glucose, triglycerides and cholesterol were analyzed using colorimetric kits (ERBA) as per manufacturer’s protocol. Estimation of Mouse Insulin (Millipore), Mouse Adiponectin (R&D Systems), were performed using ELISA kits as per manufacturer’s protocol.

### Liver cholesterol and triglyceride estimation

The estimation was done as described previously [49]. Briefly, lipid extracted from 100mg of liver tissues in hexane: isopropanol (3:2 v/v). Extracted lipids were dissolved in LPL buffer (28.75mM Pipes; 57.41 mM MgCL2.6H2O; 0.569mg/ml BSA-FFA free) with 0.1% SDS, followed by analysis from colorimetric kits (ERBA) as per manufacturer’s protocol.

### Cell culture and treatments

AML-12 hepatocytes (ATCC, Manassas, VA) were cultured in Dulbecco’s Modified Eagle Medium (DMEM), supplemented with 10% fetal bovine serum, 0.005mg/ml insulin, 0.005mg/ml transferrin, 5ng/ml selenium and 1% penicillin-streptomycin (Sigma–Aldrich, St. Louis, MO, USA). Cells were sub-cultured with 1X Trypsin-EDTA solution (Sigma–Aldrich, St. Louis, MO, USA). For all experiments cells were used at density of 5 x 10^4^ cells/24 well plate, 7 x 10^5^ cells/6 well plate or 1.2 x 10^6^ cells/60mm dish, unless otherwise mentioned. All the studies were conducted using 70-80% confluent cells, which were treated with indicated concentrations of palmitate (Sigma Aldrich, St. Louis, MO) and drugs for 24 hours. For treatment, AML-12 cells were grown in 60 mm culture dishes (Nunc, USA), and Tacrolimus and Everolimus specific dishes were pre treated with TC (20nM, 30nM) [19] and EV (20nM and 30nM) [50] 4 hours before palmitate treatment (0.25mM).

### FFA-BSA complex preparation

As described previously [39], briefly 20 % stock solution of fatty acid free BSA (A6003, Sigma-Aldrich, St. Louis, MO) was prepared in PBS. For FFA-BSA complex preparation, 1ml of 20mM aqueous solution of sodium palmitate was mixed with 3.3 ml of 20% BSA, followed by immediate addition of pre-warmed (37°C) 15.7 ml DMEM. The final solution, was sterile filtered and had concentration of 1mM palmitic acid (palmitate).

### Immunoblotting

Liver tissues (∼100mg) were homogenized using hand homogenizer ( D-1, MICCRA, Germany ) in modified lysis buffer (Tris-HCl(50mM), NaCl(150mM), EDTA (1mM), 1% IGEPAL CA-630 1% sodium deoxycholate, 0.1% SDS, 10mM DTT, 1mM PMSF, 1% protease and phosphatase inhibitor cocktails. The resulting homogenate was sonicated (Sonics Vibra-cell VCX-130 USA). After incubation at 4°C for 45 minutes, homogenized tissue lysates were centrifuge for 14000 rpm for 30 minutes at 4°C. For cell lysates, AML-12 cells were trypsinised, harvested in PBS (pH 7.4) centrifuged, and re-suspended in RIPA buffer (Sigma-Aldrich, MO, USA) for 45 minutes at 4°C. Protein estimation was done by Bradford reagent (Sigma-Aldrich, St. Louis, MO). Equal amount (50μg) of proteins from each sample were resolved on 7-15% SDS-PAGE gels (Bio-RAD), trans-blotted on poly-vinylidene difluoride (PVDF) membrane (EMD-Millipore, Billerica, MA, USA). Membranes were blocked with 5% defatted high protein milk in Tris Buffer Saline with TWEEN 20 (TBST) pH 8.0 and/or 3% Bovine Serum Albumin (BSA) in TBST pH 8.0 for two hours, and then subjected to overnight incubation with primary antibodies (anti-PPAR-γ, anti-SREBP1-c, anti-m-TOR, anti-phosphorylated m-TOR, anti-Akt, anti-phosphorylated-Akt, anti-Cytochrome P450, family 2, subfamily E, polypeptide 1 (CYP2E1), anti-cluster of differentiation 36 (CD36), anti-Bax, anti-Bcl2, anti-transforming growth factor-beta1 (TGF-β1), anti-caspase-3, Santacruz Biotech, USA), anti-SCD-1 (Cell Signaling Technology USA), anti-alpha smooth muscle actin (α-SMA) (Abcam, USA), β-Actin (Sigma-Aldrich), followed by secondary horse radish peroxidase antibodies of either anti-mouse, anti-rabbit, or anti-goat origin (Santa-Cruz Biotech, CA). Membrane visualization was done by ChemiDoc™ XRS+ (Bio-Rad, Hercules, CA, USA). Quantification of blots were done by densitometry analysis using Image Lab™ software, version 3.0, Bio-Rad (Hercules, CA, USA).

### Quantitative real-time polymerase chain reaction (qPCR)

Total RNA was isolated from snap-frozen liver tissue (∼50mg) and/or from trypsinised and harvested AML-12 cells, using RNA isolation kit as per manufacturer protocol (Qiagen Minikit,). Equal quantities of RNA were transcribed to cDNA using RT2 HT First Strand Kit (Qiagen) and subjected to real-time PCR using Roche Light Cycler 96, keeping GAPDH as control gene. Reaction volume of 20μl containing 1X Roche SYBR-Green Master Mix, Primers (forward and reverse, list given in Table -), & PCR grade water (qs) was used for all the reactions. All the data (analysed by ΔΔCt method) were normalized to control gene and represented as fold change with respect to Control group and/or un-exposed-untreated AML-12 control cells.

### Histological analysis and nile red staining

Liver histology was analyzed by histopathological expert (Dr. Subash) who was blinded to the study. Formalin fixed samples were embedded in paraffin and sectioned (10μM). Sections were stained with H&E and Nile red to examine the general morphology of liver and lipid accumulation respectively. For Nile red staining in cells, cells were grown on sterilized coverslips and incubated with indicated concentrations of palmitate alone or pretreated with tacrolimus and everolimus as mentioned. Cells were washed with PBS and fixed in methanol: acetic acid (3:1) solution overnight at 4°C. After fixation, cells were again washed and incubated in PBS containing 500ng/mL Nile red (Sigma Aldrich, St. Louis, MO) for 30 minutes at 37°C. Slides were prepared and imaging was done using Olympus FluoView FV-1000 fluorescence microscope.

### Cell viability assay

Cell viability was determined by MTT assay, as previously described [51]. After, 24 hours of indicated treatments, cells were incubated with MTT solution (0.25mg/ml in PBS) for 3 hours at 37° C. Formazan crystals formed by living cells were dissolved in DMSO and optical density was recorded at 570nm using ELISA reader (Multiskan Spectrum; Thermo Electron Corporation, USA).

### Statistical analysis

All data are expressed as mean ± S.D. Student’s t-test was used to determine significance. Analysis was performed using Primer of Biostatistics, version 4.0 (McGraw-Hill). P<0.05 was considered statistically significant.

## SUPPLEMENTARY MATERIALS FIGURE


