# Supplementary material for: Long-term administration of tacrolimus and everolimus prevents high cholesterol-high fructose-induced steatosis in C57BL/6J mice by inhibiting de-novo lipogenesis
Source: Oncotarget. 2017 Feb 8;8(69):113403–17. doi: 10.18632/oncotarget.15194 (PMC5768335; doi:10.18632/oncotarget.15194)
Supplement: Supplementary file 1 [file oncotarget-08-113403-s001.pdf]

Long-term administration of tacrolimus and everolimus prevents high cholesterol-high fructose-induced steatosis in C57BL/6J mice by inhibiting de-novo lipogenesis

SUPPLEMENTARY FIGURE

(A)

Groups	%B	%S
Control, SC	181	53
FF	116.9	19.9
TC+FF	307.2	89
EV+FF	302.6	96.2

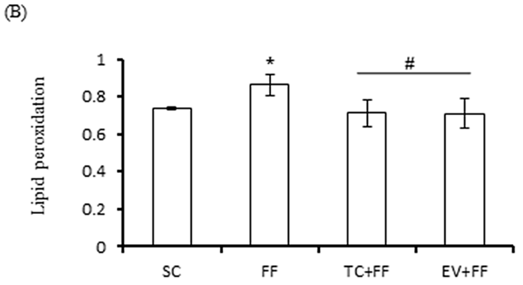

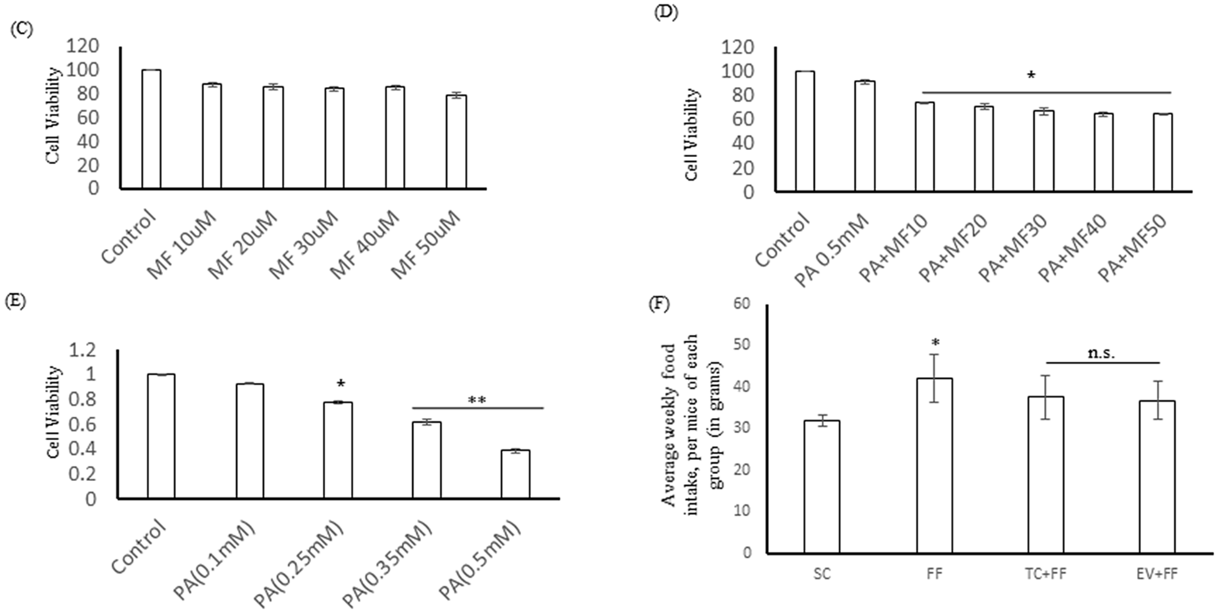

Supplementary Figure 1: A. Table shows %B (function of pancreatic beta cell) and % S (sensitivity of the insulin) of mice of respective groups, and both were also calculated by HOMA-IR calculator. FF group mice had reduced %B and %S values, which were restored by the treatment with TC and EV. **B.** Bar graph shows hepatic lipid per-oxidation values for all groups. FF group mice had increased hepatic lipid per-oxidation levels, (* indicates p< 0.05, FF versus SC), compared to SC, group while TC and EV prevented the FF induced lipid per-oxidation significantly ($^{\#}$ indicates p<0.05, TC versus FF, EV versus FF). **C.** Bar graph shows toxicity profile of MF-438, a SCD-1 inhibitor, on AML-12 cells after 24 hours of treatment, performed by MTT assay (as described earlier). Up to 50uM concentrations it has no significant cytotoxicity on AML-12 cells. **D.** Bar graph shows toxicity profile of PA (0.25mM) along with MF-438 (incubated 1 hour before, palmitate addition), on AML-12 cells. AML-12 cells co-treated with varied concentration of MF-438 (10-50uM) along with palmitate (0.25mM) had increased cell death, measured by MTT assay, as compared to cells treated with palmitate (0.25mM) only (* indicates p<0.05, palmitate (0.25mM) versus palmitate (0.25mM) + MF-438 [varied from 10 to 50 µM]). **E.** Bar graph shows toxicity profile of palmitate, in terms of fold change on 24 hours exposure on AML-12 cells, dose dependently. Palmitate at 0.1mM showed no significant cell death, but significant cell death was observed at other higher doses of palmitate. Palmitate (0.25mM) produced 20% cell death as compared to Control, un-treated cells (* indicates p<0.05, Palmitate (0.25mM) versus Control), whereas palmitate at 0.35mM and 0.5mM produced 40% and 60% cell death respectively, (*** indicates p<0.001, Palmitate (0.35mM) versus Control, Palmitate (0.5mM) versus Control). **F.** Bar graph shows average weekly food intake of per mice of each group. Food intake was measured weekly till the end of the study. SC group had significantly less food intake as compared to mice of FF group (* p<0.05, SC versus FF), however there was no significant difference regarding the food intake among other three groups which are FF versus TC, FF versus EV and TC versus EV.
